# Female gametophyte development is required for nucellar-tip degeneration during *Arabidopsis* ovule development

**DOI:** 10.1007/s00425-024-04519-7

**Published:** 2024-09-09

**Authors:** Chulmin Park, Youbong Hyun, Ji-Young Lee

**Affiliations:** 1 School of Biological Sciences, College of Natural Science, Seoul National University, Seoul, 08826 Korea; 2 Research Institute of Basic Sciences, College of Natural Sciences, Seoul National University, Seoul, 08826 Korea; 3 Research Center for Plant Plasticity, Seoul National University, Seoul, 08826 Korea; 4 Plant Genomics and Breeding Institute, Seoul National University, Seoul, 08826 Korea; 5 Plant Immunity Research Center, Seoul National University, Seoul, 00826 Korea

**Keywords:** Arabidopsis, Ovule, Female gametophyte, Nucellus, Integuments, DTA, DD13

## Abstract

Genetic ablation of the female gametophyte provides direct evidence for the existence of interregional communication during *Arabidopsis* ovule development and the importance of the female gametophyte in nucellar-tip degeneration.

The angiosperm ovule consists of three regions: the female gametophyte, the nucellus, and the integuments, all of which develop synchronously and coordinately. Previously, interregional communication enabling cooperative ovule development had been proposed; however, the evidence for these communications mostly relies on the analysis of mutant phenotypes. To provide direct evidence, we specifically ablated the *Arabidopsis* female gametophyte by expressing the diphtheria toxin fragment A (DTA) under the female gametophyte-specific *DD13* promoter and analyzed its effects on the development of the nucellus and the integuments. We found that the female gametophyte is not required for integument development or for the orientation and curvature of the ovule body, but is necessary for nucellar-tip degeneration. The results presented here provide direct evidence for communication from the female gametophyte to the nucellus and demonstrate that *Arabidopsis* ovules require interregional communication for cooperative development.

## Introduction

Ovules, the developmental precursors of seeds, represent one of the most significant innovations of Spermatophyta (seed plants) (Rudall 2021). The ovule is initiated from the placenta as a small finger-like primordium with three regions: the funiculus, the chalaza, and the nucellus along the proximal–distal axis (Skinner et al. 2004). The nucellus is the most distal region of the ovule primordium. A single inner nucellar cell differentiates into a megasporocyte, a megaspore mother cell, which undergoes meiosis to make a functional megaspore (megasporogenesis) and mitosis to produce a megagametophyte, an embryo sac (megagametogenesis), while the other nucellar cells enclose the megagametophyte. The chalaza is the central region of the ovule primordium where the integuments emerge, and they grow to cover the nucellus, leaving a small opening, the micropyle. The funiculus, the proximal region of the ovule primordium, is a vascularized stalk that links the ovule to the placenta.

**Fig. 1:**
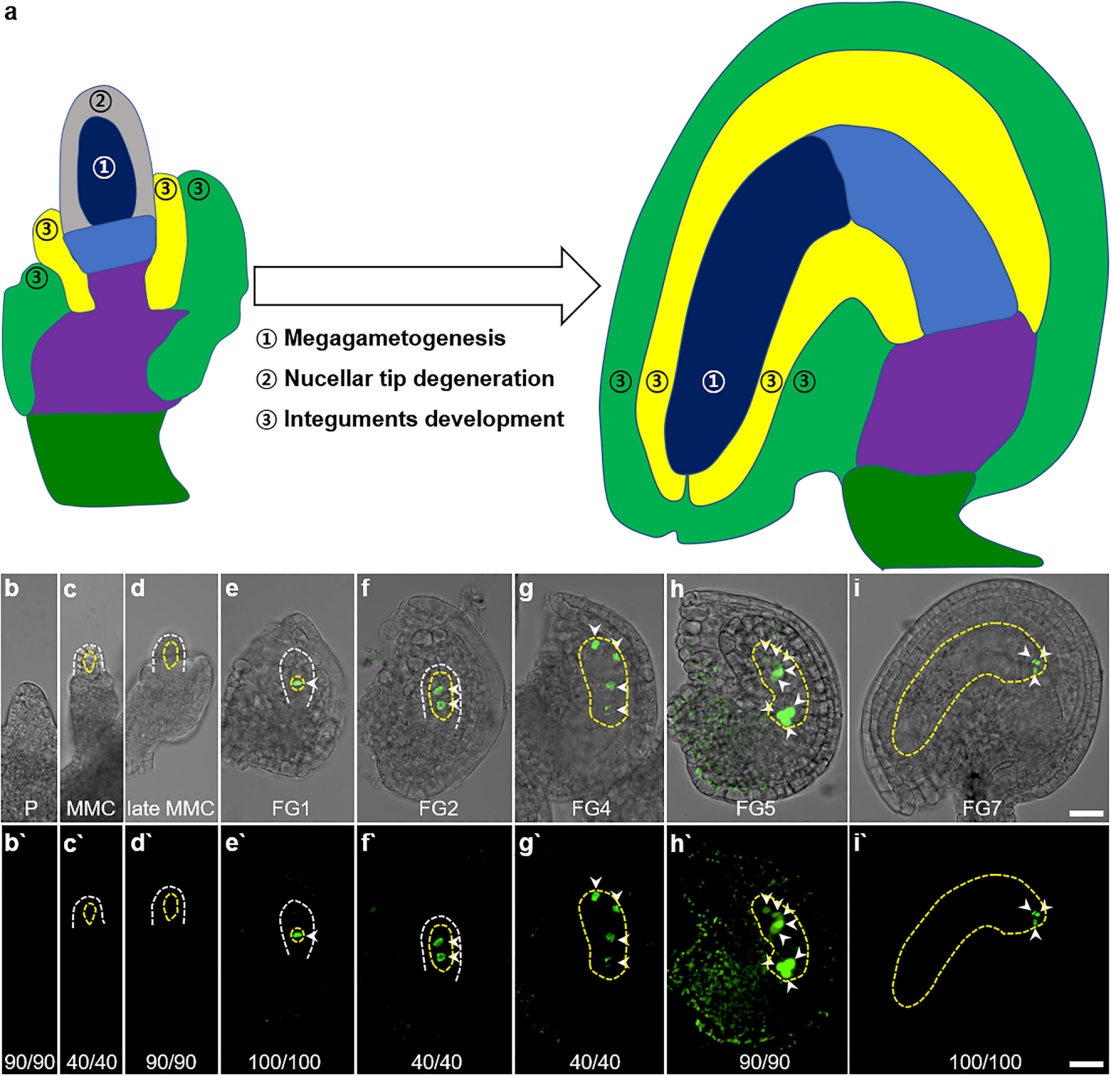
*Arabidopsis* ovule development and expression patterns of *pDD13::H2B-GFP a* Schematic representation of *Arabidopsis* ovule development. Different colors represent different tissues: dark blue (female gametophyte), gray (nucellar tip), light blue (nucellus), yellow (inner integument), light green (outer integument), violet (chalaza), and dark green (funiculus). Circled numbers indicate the regions where the processes occur. **b-i** Expression patterns of *pDD13::H2B-GFP* during *Arabidopsis* ovule development. Upper images (b-i) are merged images of bright field images and the lower GFP images (b`-i`). P (primordia), MMC (megaspore mother cell). White-dashed lines outline the outer edge of the nucellus and yellow-dashed lines encircle the megaspore mother cell and the developing female gametophyte. Arrowheads indicate female gametophyte nuclei until FG5 and antipodal cells at FG7. Scale bars: 20 μm

An ovule is established via highly dynamic and synchronous development of the female gametophyte, the nucellus, and the integuments. In the model plant *Arabidopsis*, the ovule primordium undergoes the following three major processes: 1) female gametophyte development, 2) nucellar-tip degeneration, and 3) integuments development (Fig. 1a) (Schneitz et al. 1995; Vijayan et al. 2021). 1) Female gametophyte development is a series of sequential progression. A subepidermal cell differentiates into a megaspore mother cell, which undergoes meiosis to produce four megaspores (megasporogenesis). Only the chalazal megaspore survives, expands, and curves, accompanied by three rounds of mitosis without cytokinesis, followed by nuclear migration and cellularization. These eventually give rise to the mature curved female gametophyte with eight nuclei in seven cells (megagametogenesis). The female gametophyte expands nine-fold in volume during this process and bends gynapically (Vijayan et al. 2021). 2) The single-layered nucellar tip and the nucellar cells surrounding the female gametophyte degenerate through vacuolar cell death in concert with female gametophyte development, providing space for the expanding embryo sac (Wang et al. 2021). 3) The inner and outer integuments emerge from the chalazal epidermis, undergo planar or laminar growth to form sheet-like lateral organs, asymmetrically surround the female gametophyte to create the micropyle on the gynapical side, and eventually shape the curved ovule body. These coordinated processes establish a campylotropous tenuinucellate bitegmic ovule (Robinson-Beers et al. 1992; Shamrov 2018).

In addition to synchronous and coordinated ovule development, molecular and genetic analyses of genes controlling ovule development also support the idea that ovule development is a cooperative process requiring interregional communication among the female gametophyte, the nucellus, and the integuments (Gasser et al. 1998; Gross-Hardt et al. 2002; Grossniklaus and Schneitz 1998). However, it remains elusive which processes exactly require interregional communication. Current studies of gene expression patterns and their mutant phenotypes during ovule development cannot fully address interregional communication. Therefore, independent experiments are necessary to demonstrate the presence of interregional communication in specific processes during ovule development.

Here, we elucidated the roles of the female gametophyte in cooperative ovule development. To eliminate the effects of the female gametophyte on nucellus and integuments development, we specifically ablated the *Arabidopsis* female gametophyte by expressing the diphtheria toxin fragment A (DTA) under the female gametophyte-specific *DD13* promoter (Steffen et al. 2007; Yu et al. 2016; Yuan et al. 2016) and analyzed its effects on nucellus and integuments development. We found that the female gametophyte is not required for integuments development nor the orientation and curvature of the ovule body, but it is necessary for nucellar-tip degeneration. These results demonstrate that *Arabidopsis* ovule development is cooperative and requires communication among the female gametophyte, the nucellus, and the integuments, and, particularly, the female gametophyte plays a crucial role in nucellar-tip degeneration.

## Materials and methods

### Plant materials and growth conditions

*Arabidopsis* (*Arabidopsis thaliana*) ecotype Columbia (Col-0) was used. *pDD13::H2B-GFP* and *pDR5revV2::n3GFP – pTCSn::ntdTomato* were previously described (Smet et al. 2019; Yu et al. 2016). The plants were grown under a 16-h–light/8-h–dark cycle at 22°–23 °C in a plant growth chamber.

### Construction of transgenic plants

Gateway cloning technology (Invitrogen) was used for DNA manipulations. The *DD13* promoter region (Yu et al. 2016) was amplified from *Arabidopsis* Col-0 genomic DNA by PCR using pDD13-F-attB4 (GGGGACAACTTTGTATAGAAAAGTTGCGatctttttgataaatgagagtactatattgtg) and pDD13-R-attB1r (GGGGACTGCTTTTTTGTACAAACTTGCTCTCAAAATCTGCATATATCTTTTTAATGAC), and inserted into pDONR P4_P1R via a BP reaction. The *DTA* CDS was amplified using DTA-F (caccATGGATCCTGATGATGTTGTT) and DTA-R (TTAGAGCTTTAAATCTCTGTA G), and cloned into pENTR/D-TOPO. *pDD13::DTA* was constructed into dpGreen-BarT by means of Multisite Gateway LR recombination and transformed into *Agrobacterium* GV3101 with pSOUP for *Arabidopsis* transformation via floral dipping. Transgenic plants were selected with a 2,000-fold diluted Basta (Bayer Crop Science) solution on soil.

### Phenotype and segregation analysis

Morphologic observations of rosettes and inflorescences were conducted using an optical camera (Nikon D7000). A Zeiss Stemi 508 stereomicroscope coupled with an Axiocam 208 color camera was used to observe and analyze the phenotypes of siliques and seeds. For quantitative analysis of fertilization rates, siliques were dissected under the stereomicroscope, and fertilized and unfertilized ovules were manually counted. Three and five siliques from individual plants were analyzed in T1 and T2, respectively. For segregation analysis, the basta resistance of approximately a hundred seeds on MS plates supplemented with 10 ug/ml basta was examined.

### Microscopy

For DIC microscopy images of developing ovules, ovules were cleared at the mentioned stages in a chloral hydrate solution (chloral hydrate/water/glycerol, 8/3/1, w/v/v) and imaged with a ZEISS A1 microscope coupled to an Axiocam 712 color camera. For confocal images of developing ovules, ovules were mounted in a 10% glycerol solution and imaged directly using a Nikon ECLIPSE Ti2 laser scanning confocal microscope with excitation/detection wavelengths of 488 nm/493 to 547 nm for GFP, and 552 nm/557 to 737 nm for autofluorescence.

## Results

### The DD13 promoter is exclusively active in female gametophytic cells

To investigate the roles of the female gametophyte in interregional communication during ovule development, we searched for a promoter that is exclusively active in the female gametophyte. As a result, we found that the *DD13* promoter is ideal for this purpose. *DD13* (At3g59260), encoding a pirin-like protein, was identified as an antipodal cell-specific gene in the mature ovule (Steffen et al. 2007), and the *DD13* promoter has been used to generate an antipodal cell marker (Yu et al. 2016; Yuan et al. 2016). When we examined the expression of histone *H2B-GFP* driven by *DD13* promoter (*pDD13::H2B-GFP*) (Yu et al. 2016) throughout *Arabidopsis* ovule development, we confirmed that the *pDD13::H2B-GFP* signal is only detected in the antipodal cells in the mature ovule (Fig. 1i). However, in earlier stages of ovule development, we observed previously unreported *pDD13::H2B-GFP* signals from the functional megaspore (Fig. 1e) and the subsequent female gametophytic nuclei (Fig. 1f–h). Since we did not observe any *pDD13::H2B-GFP* signals in the ovule primordia and the megaspore mother cell (Fig. 1b-d), we concluded that the *DD13* promoter is active not only in the antipodal cells after the cellularization of the female gametophyte but also in every female gametophyte nucleus during the syncytial stage (Fig. 1e-i). These findings suggest that the *DD13* promoter can be used as an undifferentiated syncytial stage marker of the female gametophyte, in addition to an antipodal cell marker.

### DD13 promoter-driven DTA induces defects in the female gametophyte

**Fig. 2:**
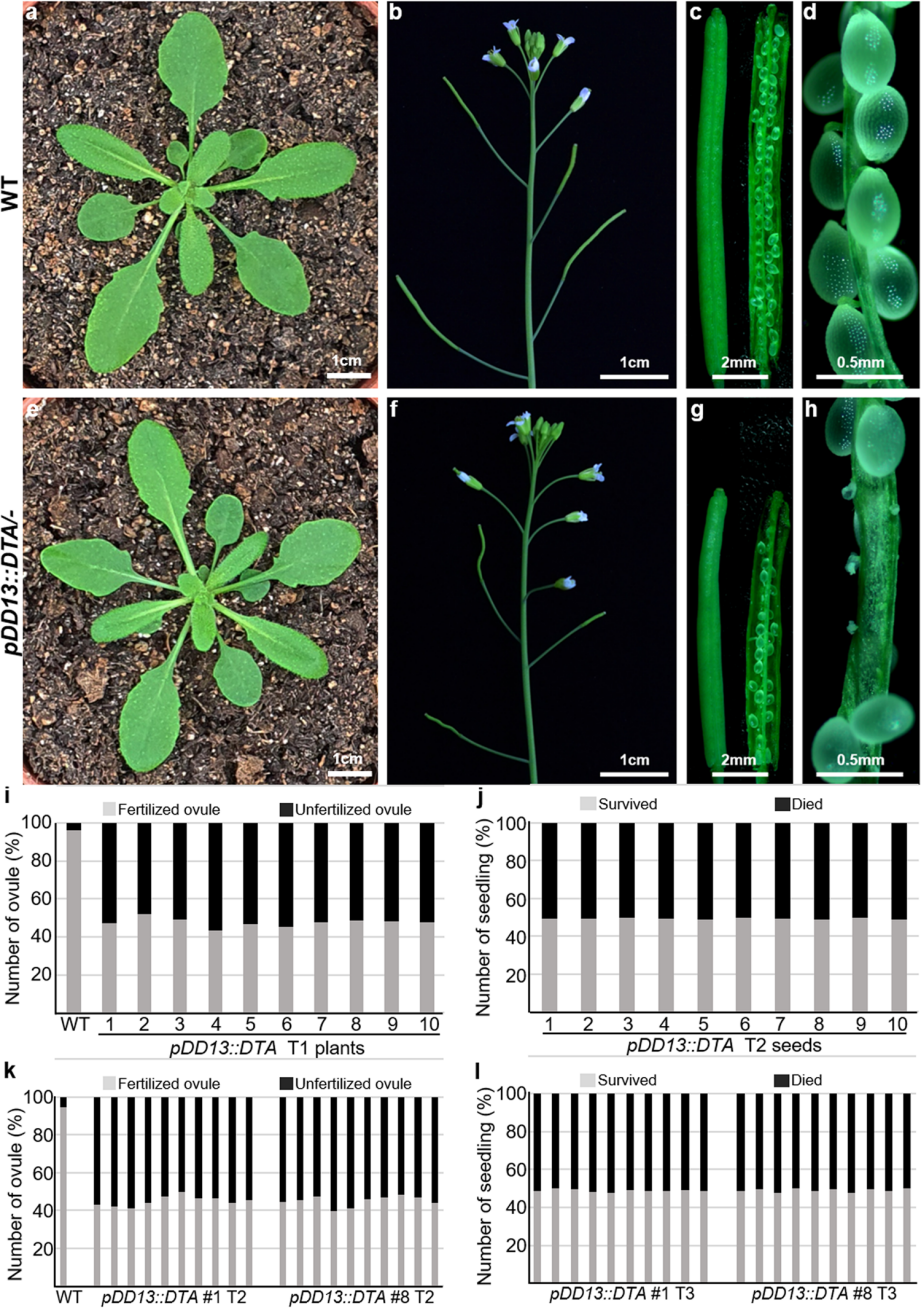
Analysis of phenotype and segregation rate of *pDD13::DTA* transgenic plants Phenotypes of rosettes (**a**, **e**), inflorescences (**b**, **f**), siliques (**c**, **g**), and seeds (**d**, **h**) in the wild-type (**a**-**d**) and *pDD13::DTA/-* (**e**–**h**) plants. **i** Fertilization rates of the wild-type and ten-independent *pDD13::DTA* T1 plants. **j** Segregation ratio of *pDD13::DTA* T2 seeds from ten-independent T1 plants on agar plates containing basta. **k** Fertilization rates of the wild-type and ten-individual plants of two representative T2 lines. **l** Segregation ratio of *pDD13::DTA* T3 seeds from ten-individual plants of two representative T2 lines

To examine the roles of the female gametophyte in ovule development through specific ablation of the female gametophyte, we generated *pDD13::DTA* transgenic plants expressing DTA under the *DD13* promoter. DTA kills cells by blocking protein synthesis (Collier 1967) and the encoding gene has been used to ablate specific tissues in plants (Day et al. 1995; Nilsson et al. 1998; Tsugeki and Fedoroff 1999; Weijers et al. 2003). We analyzed 10 independent transgenic T1 plants and found that *pDD13::DTA* plants had no defects in any organs besides siliques (Fig. 2a–h). Siliques of *pDD13::DTA* plants were shorter than those of the wild-type, and the fertilization rates were around 50% (Fig. 2b–d, f–i). To test whether the phenotype is transmitted sporophytically or gametophytically, we checked the segregation ratio in T2 plants. If the seedlings have the transgene, they could survive on the basta plate because they carry a basta-resistant gene used as a selection marker. All T2 plants from 10 independent T1 plants showed around 50% survival rates, indicating a 1:1 segregation ratio (Fig. 2j). Similarly, 50% fertilization rates and a 1:1 segregation ratio were observed in T2 siliques and T3 seedlings, respectively (Fig. 2k, l). It is noteworthy that all tested-T2 plants were hemizygous for the transgene, and we could not obtain any homozygous of transgene based on the segregation ratio in T3 plants (Fig. 2l). These segregation distortion (SD) results indicate that the *pDD13::DTA* transgene fails to transmit through the female gametophyte by triggering gametophytic lethality (Christensen et al. 1998; Drews and Koltunow 2011; Howden et al. 1998).

**Fig. 3:**
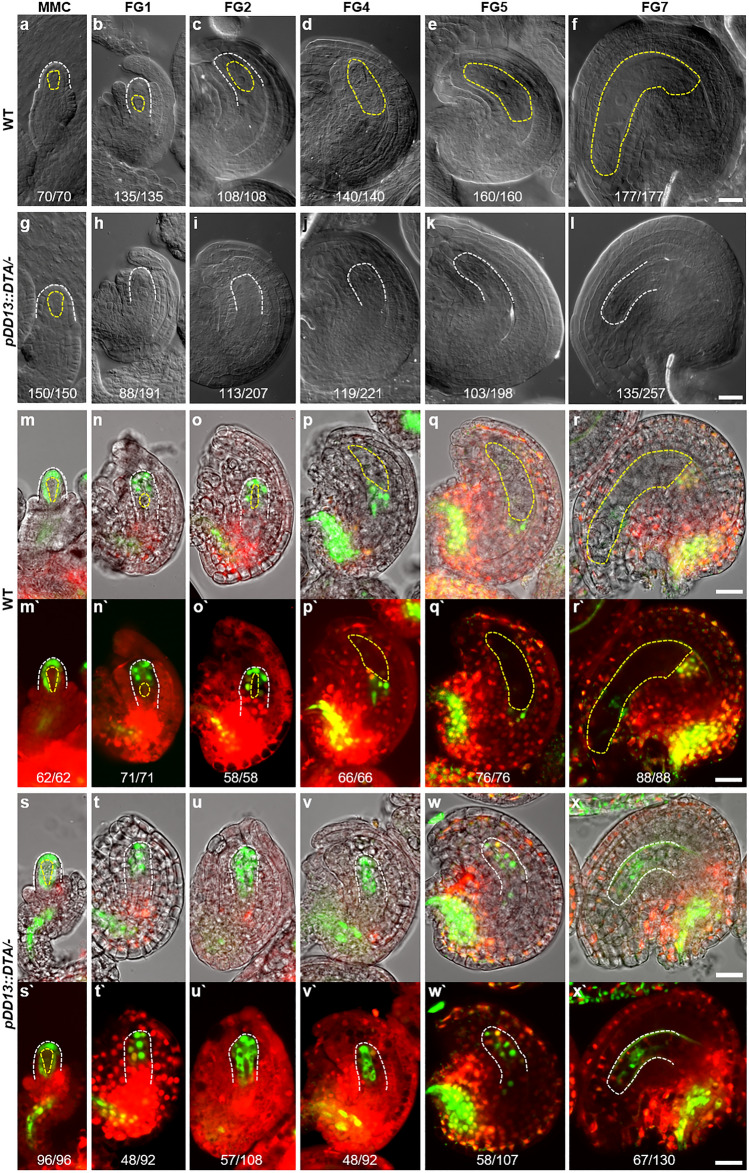
Cytologic analysis and auxin response of developing ovules in the wild-type and *pDD13::DTA/-*plants **a-l** DIC microscopy images of the wild-type (**a**-**f**) and *pDD13::DTA/-* (**g**-**l**) ovules at the indicated stages. **m**-**x** Confocal images of the wild-type **(m**-**r)** and *pDD13::DTA/-* (**s**-**x**) plants carrying an auxin-signaling reporter (*pDR5revV2::n3GFP*). Upper images (**m**-**x**) are merged images of the bright field and the lower fluorescence images (**m`**-**x`**). MMC, megaspore mother cell. White-dashed lines outline the outer edge of the nucellus and yellow-dashed lines encircle the megaspore mother cell and the developing female gametophyte. Scale bars: 20 μm

Next, we analyzed the ovule phenotypes of *pDD13::DTA* plants and found that around 50% of ovules of *pDD13::DTA* plants did not develop female gametophytes, and the others were identical to the wild-type ovules (Fig. 3f, l). Since these transgenic plants were hemizygous, *pDD13::DTA* likely triggered the ablation of the female gametophytes.

### The ablation of the female gametophyte induces defects in nucellar-tip degeneration

After confirming that *pDD13::DTA* specifically ablates the female gametophyte, we investigated the roles of the female gametophyte in ovule development. Between wild-type and female gametophyte-ablated mature ovules, there was no difference in asymmetric integuments development to establish curvature and micropyle (Fig. 3f, l). However, the nucellar tip remained in around 50% of ovules from *pDD13::*DTA hemizygous plants, in contrast to wild-type mature ovules where the nucellar tip degenerated (Fig. 3f, l).

To understand the roles of the female gametophyte in nucellar-tip degeneration in more detail, we sequentially analyzed ovule development after functional megaspore formation in both wild-type and female gametophyte-ablated ovules. In the wild-type ovule, the haploid functional megaspore undergoes three rounds of mitotic divisions, enlarges, and becomes vacuolated (Fig. 3a–f, m–r). Concurrently with this megagametogenesis, the nucellar tip degenerates, providing space for the expanding female gametophyte (Lu and Magnani 2018). In contrast to the wild-type, around 50% of ovules from *pDD13::DTA* hemizygous plants retained the nucellar tip even though the megagametophyte was absent (Fig. 3g–l, s–x).

To confirm these results, we examined the auxin response, which is high in the degenerating nucellar cells and plays an important role in nucellar-tip degeneration (Wang et al. 2021). In wild-type ovules, the auxin response was strong in the nucellar tip until FG2 (Fig. 3m–o), and as nucellar cell degeneration progressed from the distal nucellar tip toward the proximal nucellus, the auxin response also moved accordingly, as previously reported (Fig. 3p–r) (Wang et al. 2021). In contrast, in around 50% of ovules from *pDD13::DTA* hemizygous plants, the auxin response persisted in the distal nucellar cells throughout ovule development (Fig. 3s–x), supporting the notion that the distal cells with high auxin responses are the nucellar-tip cells that did not degenerate.

These data suggest that the female gametophyte is not required for integument development but is necessary for nucellar-tip degeneration.

## Discussion

Ovule development is a highly dynamic and synchronous process involving interactions among the female gametophyte, the nucellus, and the integuments. These processes and their interactions have mainly been studied based on mutant phenotypes with defective ovule development. Here, we provide direct evidence for the existence of interregional communication during ovule development. By utilizing a genetic tool that specifically ablates the female gametophyte, we demonstrate that the female gametophyte is not required for integument development nor the orientation and curvature of the ovule body; however, it is necessary for nucellar-tip degeneration.

Although several genes regulating ovule development have been identified, it remains difficult to determine the effect of the female gametophyte on integument development based solely on mutant phenotypes. For example, both *SPL*/*NZZ* and *WUS* are expressed in the nucellar tip, and their mutants exhibit defects in megasporogenesis. However, these mutants display different integument phenotypes: while the *spl*/*nzz* mutant develops normal integuments, the *wus* mutant lacks integuments (Gross-Hardt et al. 2002; Lieber et al. 2011; Vijayan et al. 2021). The data presented here provide a clear answer to this question: the female gametophyte is not required for integument development.

Regarding cooperative ovule development, *INNER NO OUTER* (*INO*) is one of the most studied genes. *INO* is expressed in the outer integument, and its mutant ovule exhibits defects in megagametogenesis (blocked at the mono-nuclear embryo sac stage) and nucellar-tip degeneration. This implies that *INO* regulates megagametogenesis and nucellar tip degeneration in a non-cell-autonomous manner (Baker et al. 1997; Schneitz et al. 1997; Vijayan et al. 2021). However, it remains unclear whether *INO* regulates nucellar-tip degeneration in parallel with or through the female gametophyte. Here, we found that the female gametophyte is necessary for nucellar-tip degeneration, favoring the idea that *INO* indirectly regulates nucellar-tip degeneration through the female gametophyte.

Recently, Wang et al. found that the polar transport of distal maternal auxin into the nucellar tip via auxin efflux carriers is crucial for nucellar-tip degeneration (Wang et al. 2021). Interestingly, we found that in the female gametophyte-ablated ovule, auxin signaling remains strong in the nucellar tip, yet the nucellar tip does not degenerate. This suggests that both auxin signaling and the presence of the female gametophyte are necessary for nucellar-tip degeneration.

The results presented here substantiate the presence of interregional communication among the female gametophyte, the nucellus, and the integuments, which enables cooperative ovule development, highlighting the importance of the female gametophyte for nucellar-tip degeneration.

## Declarations

### Data availability

All relevant data can be found within the manuscript.
